# Supplementary material for: Switching Heavy Chain Constant Domains Denatures the Paratope 3D Architecture of Influenza Monoclonal Antibodies
Source: Pathogens. 2022 Dec 28;12(1):51. doi: 10.3390/pathogens12010051 (PMC9865026; doi:10.3390/pathogens12010051)
Supplement: Supplementary file 1 [file pathogens-12-00051-s001.zip › pathogens-2022523-supplementary.pdf]

Variable and constant domain sequences used to genetically engineer VR analogous IgG variants

Human IgG1 CH1, Hinge, CH2-CH3

ASTKGPSVFPLAPSSKSTSGGTAALGCLVKDYFPEPVTVSWNSGALTSGVHTFPAVLQSSGLYSLSSVVTVPSSSLG
TQTYICNVNHKPSNTKVDKKVEPKSCDKTHTCPPCPAPELLGGPSVFLFPPKPKDTLMISRTPEVTCVVVDVSHEDPE
VKFNWYVDGVEVHNAKTKPREEQYNSTYRVVSVLTVLHQDWLNGKEYKCKVSNKALPAPIEKTISKAKGQPREPQV
YTLPPSRDELTKNQVSLTCLVKGFYPSDIAVEWESNGQPENNYKTTPPVLDSDGSFFLYSKLTVDKSRWQQGNVFSC
SVMHEALHNHYTQKSLSLSPGK

Human IgG4 CH1, Hinge, CH2-CH3

ASTKGPSVFPLAPCSRSTSESTAALGCLVKDYFPEPVTVSWNSGALTSGVHTFPAVLQSSGLYSLSSVVTVPSSSLG
TKTYTCNVDHKPSNTKVDKRVESKYGPPCPSCPAPEFLGGPSVFLFPPKPKDTLMISRTPEVTCVVVDVSQEDPEVQ
FNWYVDGVEVHNAKTKPREEQFNSTYRVVSVLTVLHQDWLNGKEYKCKVSNKGLPSSIEKTISKAKGQPREPQVYTL
PPSQEEMTKNQVSLTCLVKGFYPSDIAVEWESNGQPENNYKTTPPVLDSDGSFFLYSRLTVDKSRWQEGNVFSCSV
MHEALHNHYTQKSLSLSLGK

Mouse IgG1 CH1, Hinge, CH2-CH3

AKTTPPSVYPLAPGSAAQTNSMVTLGCLVKGYFPEPVTVTWNSGSLSSGVHTFPAVLQSDLYTLSSSVTVPSSTWPS
ETVTCNVAHPASSTKVDKKIVPRDCGCKPCICTVPEVSSVFIFPPKPKDVLTITLTPKVTCVVVDISKDDPEVQFSWFV
DDVEVHTAQTQPREEQFNSTFRSVSELPIMHQDWLNGKEFKCRVNSAAFPAPIEKTISKTKGRPKAPQVYTIPPPKEQ
MAKDKVSLTCMITDFFPEDITVEWQWNGQPAENYKNTQPIMDTDGSYFVYSKLNVQKSNWEAGNTFTCSVLHEGLH
NHHTEKSLSHSPGK

Mouse IgG2a CH1, Hinge, CH2-CH3

AKTTAPSVYPLAPVCGDTTGSSVTLGCLVKGYFPEPVTLTWNSGSLSSGVHTFPAVLQSDLYTLSSSVTVTSSTWPS
QSITCNVAHPASSTKVDKKIEPRGPTIKPCPPCKCPAPNLLGGPSVFIFPPKIKDVLMISLSPIVTCVVVDVSEDDPDVQI
SWFVNNVEVHTAQTQTHREDYNSTLRVVSALPIQHQDWMSGKEFKCKVNNKDLPAPIERTISKPKGSVRAPQVYVLP
PPEEEMTKKQVTLTCMVTDFMPEDIYVEWTNNGKTELNYKNTEPVLDSDGSYFMYSKLRVEKKNWVERNSYSCSVV
HEGLHNHHTTKSFSRTPGK

Mouse J-Chain

TGDDEATILADNKCMCTRVTSRIIPSTEDPNEDIVERNIRIVVPLNNRENISDPTSPLRRNFVYHLSDVCKKCDPVEVEL
EDQVVTATQSNICNEDDGVPETCYMYDRNKCYTTMVPLRYHGETKMVQAALTPDSCYPD

Mouse IgA CH1, Hinge, CH2-CH3

ESARNPTIYPLTLPPALSSDPVIIGCLIHDYFPSGTMNVTWGKSGKDITTVNFPPALASGGRYTMSSQLTLPAVECPEG
ESVKCSVQHDSNPVQELDVNCSGPTPPPPITIPSCQPSLSLQRPALEDLLLGSDASITCTLNGLRNPEGAVFTWEPST
GKDAVQKKAVQNSCGCYSVSSVLPGCAERWNSGASFKCTVTHPESGTLTGTIAKVTVNTFPPQVHLLPPPSEELALN
ELLSLTCLVRAFNPKEVLVRWLHGNEELSPESYLVFEPLKEPGEGATTYLVTSVLRVSAETWKQGDQYSCMVGHEAL
PMNFTQKTIDRLSGKPTNVSVSVIMSEGDGICY

F045-092

HC variable region

EVQLVESGAEVKKPGSSVKVSCRASGTFYKYAINWVRQAPGQGLEWMGGIIPFFGTTNYAQKFQGRLTITADGSTNT
AYMQLDSLRSEDTAVYYCAGPSITESHYCLDCAAKDYYYGLDVWGQGTTVTVSS

LC

QSVLTQPPSASGTPGQSVTISCSGSRSNIGGNTVNWYQHLPGMAPKLLIYSSNQRSSGVPDRFSGSKSGTSASLAIS
GLQSEDDADYYCASWDDSLNGVVFGGGTKLTVLGQPKAAPSVTLFPPSSEELQANKATLVCLISDFYPGAVTVAWKA
DSSPVKAGVETTTPSKQSNNKYAASSYLSLTPEQWKSHRSYSCQVTHEGSTVEKTVAPTECS

CR6261 variable regions

HC variable region

EVQLVESGAEVKKPGSSVKVSCKASGGPFRSYAISWVRQAPGQGPEWMGGIIPIFGTTKYAPKFQGRVTITADDFAG
TVYMELSSLRSEDTAMYYCAKHMGYQVRETMDVWGKGTTVTVSS

LC

QSVLTQPPSVSAAPGQKVTISCSGSSSNIGNDYVSWYQQLPGTAPKLLIYDNNKRPSGIPDRFSGSKSGTSATLGITG
LQTGDEANYYCATWDRRPTAYVVFGGGTKLTVLGAAAGQPKAAPSVTLFPPSSEELQANKATLVCLISDFYPGAVTV
AWKADSSPVKAGVETTTPSKQSNNKYAASSYLSLTPEQWKSHRSYSCQVTHEGSTVEKTVAPTECS

FI6

HC variable region

QVQLVESGGGVVQPGRSLRLSCAASGFTFSTYAMHWVRQAPGKGLEWVAVISYDANYKYYADSVKGRFTISRDNSK
NTLYLQMNSLRAEDTAVYYCAKDSQLRSLLYFEWLSQGYFDYWGQGTLVTVSS

LC

DIVMTQSPDSLAVSLGERATINCKSSQSVTFNYKNYLAWYQQKPGQPPKLLIYWASTRESGVPDRFSGSGSGTDFTL
TISSLQAEDVAVYYCQQHYRTPPTFGQGTKVEIKRTVAAPSVFIFPPSDEQLKSGTASVVCLLNNFYPREAKVQWKVD
NALQSGNSQESVTEQDSKDSTYSLSSTLTLSKADYEKHKVYACEVTHQGLSSPVTKSFNRGEC

5J8 variable regions

HC variable region

EVQLVESGPGLVKPSDILSLTCAVSGYSISSNYYWGWIRQPPGKGLEWIGSIYHSGSTYYKPSLESRLGISVDTSKNQ
FSLKLSFVSAADTAVYYCARHVRSGYPDTAYYFDKWGQGTLVTVSS

LC

SYVLTQPPSVSVAPGETARISCGGNNIGTKVLHWYQQTPGQAPVLVVYDDSDRPSGIPERFSGSNSGNTATLTISRV
EVGDEADYYCQVWDISTDQAVFGGGTKLTVLGQPKAAPSVTLFPPSSEELQANKATLVCLISDFYPGAVTVAWKADS
SPVKAGVETTTPSKQSNNKYAASSYLSLTPEQWKSHRSYSCQVTHEGSTVEKTVAPTECS

MAB61.1.3

Mouse IgG1, CH1, Hinge, CH2-CH3

VKLQESGAELARPGASVKMSCKASGYTFTTYTIHWIKQRPGQGLEWIGYINPSSVYTNYNQRFKDKATLTRDRSSNT
ANIHLSSLTSDDSAVYYCVREGEVPYWGQGTTVTVSSAKTTPPSVYPLAPGSAAQTNSMVTLGCLVKGYFPEPVTVT
WNSGSLSSGVHTFPAVLQSDLYTLSSSVTVPSSPRPSETVTCNVAHPASSTKVDKKIVPRDCGCKPCICTVPEVSSVF
IFPPKPKDTLLITVTPKVTCVVVDISKDDPEVQFSWFVDNVEVHTAQTQPREEQFNSTFRVVSALPIMHQDWLNGKEF
KCRVNSAAFPAPIEKTISKTKGKPRAPQVYTIPPPKEQMAKDKVSLTCMITDFFPEDITVEWQSDGQAPENYKNTQPIM
DTDGSYFVYSKLNVQKSNWEAGNTFTCSVLHEGLHNHHTEKSLSH

LC

KCAHTVSKSMSMSVGERVTLTCKASENVVTYVSWYQQKPEQSPKLLIYGASNRYTGVPDRFTGSGSATDFTLTISSV
QAEDLADYHCGQGYSYPYTFGGGTKLEIKRADAAPTVSIFPPSSEQLTSGGASVVCFLNNFYPKDINVKWKIDGSER
QNGVLNSWTDQDSKDSTYSMSSTLTLTKDEYERHNSYTCEATHKTSTSPIVKSFNRNEC

MAB231

Mouse IgG2a, CH1, Hinge, CH2-CH3

EVKLQESGGGLVQPGGSLKLSCATSGFTFSDYYMYWVRQTPEKRLEWVAYISNGGGSTYYPDTVKGRFTISRDNAK
NTLYLQMSRLKSEDTAMYYCARHGGYYAMDYWGQGTTVTVSSAKTTAPSVYPLAPVCGDTTGSSVTLGCLVKGYFP
EPVTLTWNSGSLSSGVHTFPAVLQSDLYTLSSSVTVTSSTWPSQSITCNVAHPASSTKVDKKIEPRGPTIKPCPPCKC
PAPNLLGGPSVFIFPPKIKDVLMISLSPIVTCVVVDVSEDDPDVQISWFVNNVEVHTAQTQTHREDYNSTLRVVSALPIQ
HQDWMSGKEFKCKVNNKDLPAPIERTISKPKGSVRAPQVYVLPPPEEEMTKKQVTLTCMVTDFMPEDIYVEWTNNG
KTELNYKNTEPVLDSDGSYFMYSKLRVEKKNWVERNSYSCSVVHEGLHNHHTTKSFSR

LC

DIVLTQSPSSLSASLGDTITITCHASQNINVWLSWYQQKPGNIPKLLIYKASNLHTGVPSRFSGSGSGTGFTLTISSLQP
EDIATYYCQQGQSYPLTFGGGTKLEIKRADAAPTVSIFPPSSEQLTSGGASVVCFLNNFYPKDINVKWKIDGSERQNG
VLNSWTDQDSKDSTYSMSSTLTLTKDEYERHNSYTCEATHKTSTSPIVKSFNRNEC

B12

Human IgG, CH1, Hinge, CH2-CH3

QVQLVQSGAEVKKPGASVKVSCQASGYRFSNFVIHWVRQAPGQRFEWMGWINPYNGNKEFSAKFQDRVTFTADTS
ANTAYMELRSLRSADTAVYYCARVGPYSWDDSPQDNYYMDVWGKGTTVIVSSASTKGPSVFPLAPSSKSTSGGTAA
LGCLVKDYFPEPVTVSWNSGALTSGVHTFPAVLQSSGLYSLSSVVTVPSSSLGTQTYICNVNHKPSNTKVDKKAEPK
SCDKTHTCPPCPAPELLGGPSVFLFPPKPKDTLMISRTPEVTCVVVDVSHEDPEVKFNWYVDGVEVHNAKTKPREEQ
YNSTYRVVSVLTVLHQDWLNGKEYKCKVSNKALPAPIEKTISKAKGQPREPQVYTLPPSRDELTKNQVSLTCLVKGFY
PSDIAVEWESNGQPENNYKTTPPVLDSDGSFFLYSKLTVDKSRWQQGNVFSCSVMHEALHNHYTQKSLSLSPGK

LC

EIVLTQSPGTLSLSPGERATFSCRSSHSIRSRRVAWYQHKPGQAPRLVIHGVSNRASGISDRFSGSGSGTDFTLTITR
VEPEDFALYYCQVYGASSYTFGQGTKLERKRTVAAPSVFIFPPSDEQLKSGTASVVCLLNNFYPREAKVQWKVDNAL
QSGNSQESVTEQDSKDSTYSLSSTLTLSKADYEKHKVYACEVTHQGLRSPVTKSFNRGEC

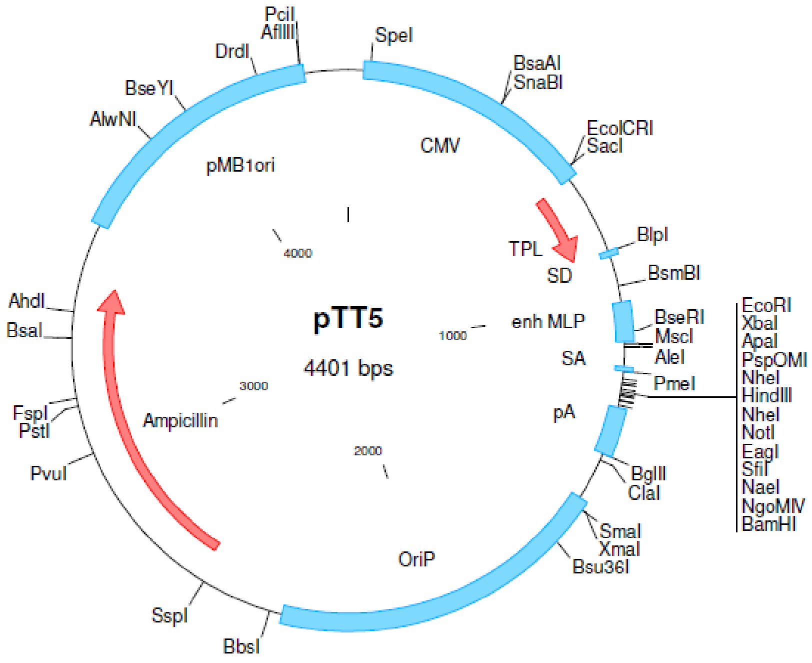

Figure S1. Plasmid map for a pTT5 expression vector.

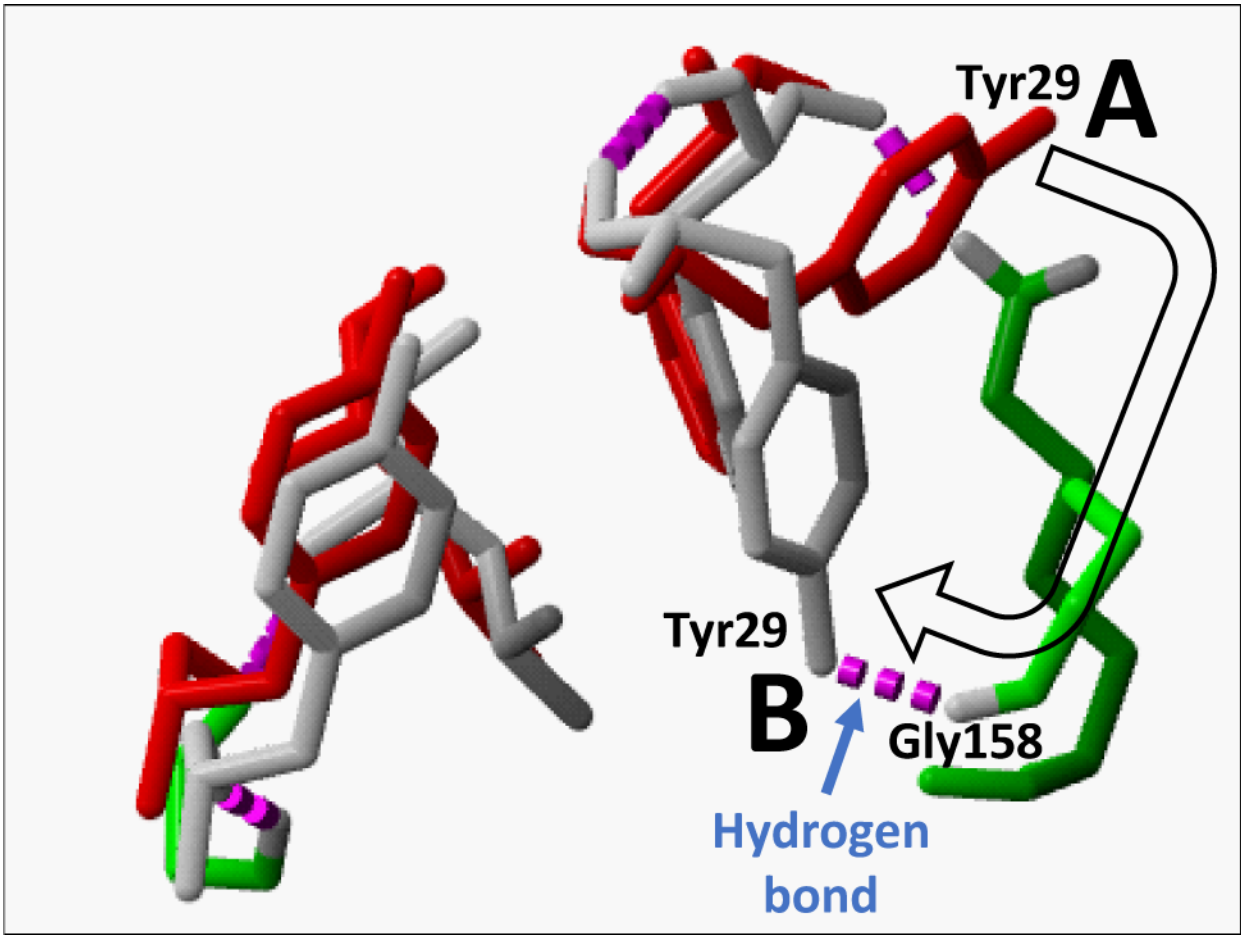

Figure S2. F045-092 monoclonal antibody uses the induced fit mechanism to bind HA. Depicted are exposed corresponding amino acid residues (as sticks) of structurally aligned crystal structures of HA-unbound Fab (red) and HA-(green)-bound Fab (grey). The orientation of Tyr29 in unbound Fab is position A. The orientation of Tyr29 when the Fab binds HA is position B. The distance (black arrow) covered by Tyr29 to move from position A to position B to form a hydrogen bond (magenta) with Gly158 on HA is 8.42 Angstroms (A). The above statement does not say that the hydrogen bond can form between position A and B given that 8.42 A is outside the hydrogen bond forming distance of < 4 A.

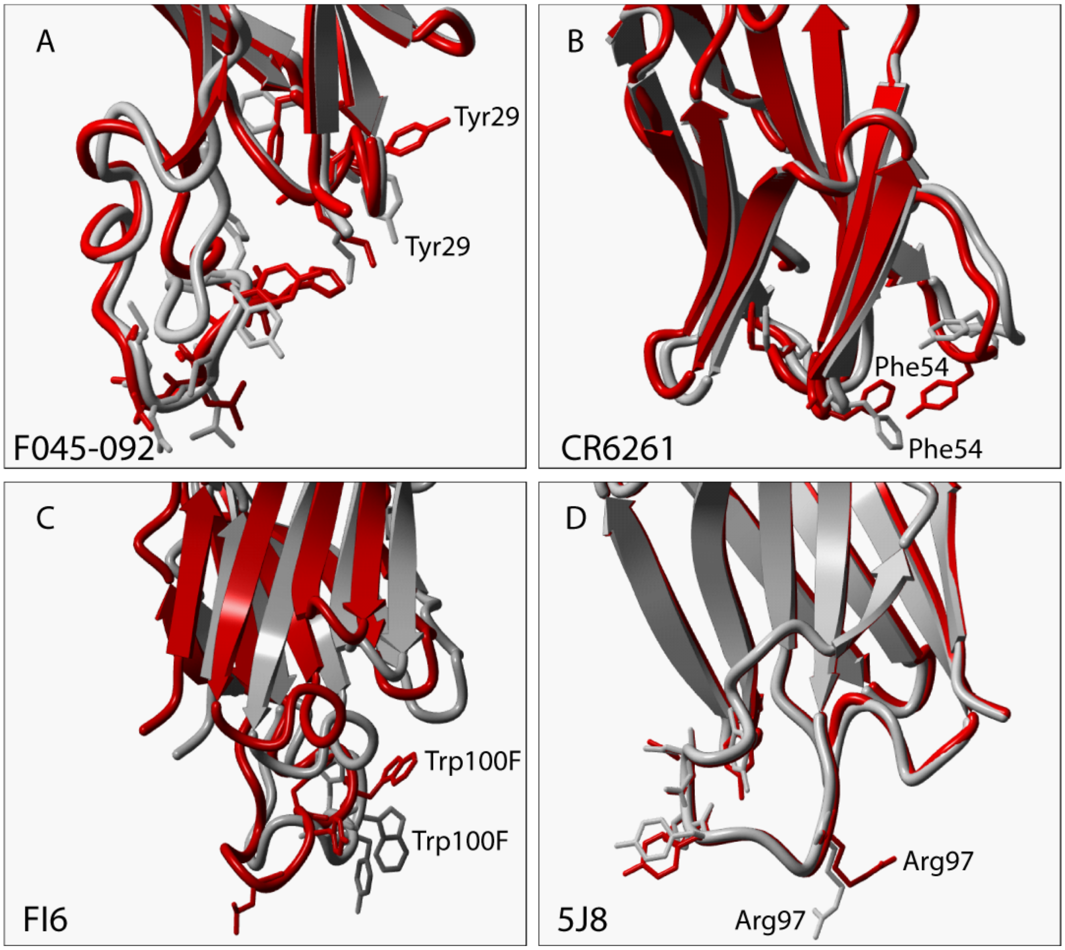

Figure S3. Structural alignment of predicted human IgG1 (grey) and human IgG1 containing a mouse IgG1 hinge (red). Differences in both location and orientation of corresponding paratope amino acid residues were observed. A: F045-092, B: CR6261, C: FI6 and D: 5J8).

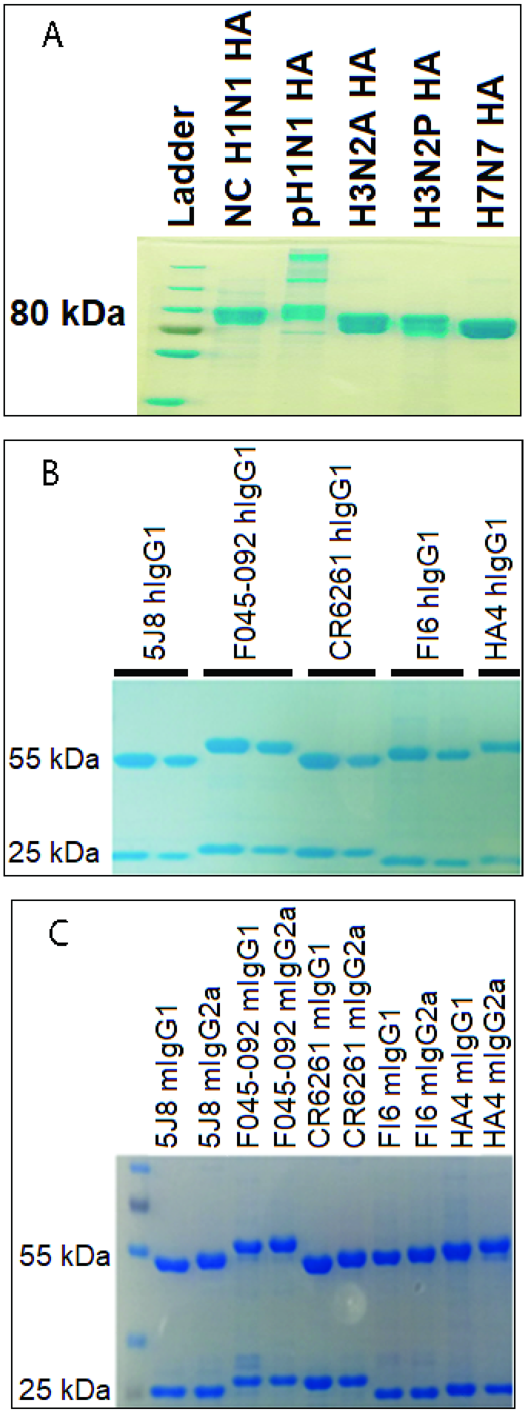
A
Ladder
NC H1N1 HA
pH1N1 HA
H3N2A HA
H3N2P HA
H7N7 HA
80 kDa
B
5J8 hIgG1
F045-092 hIgG1
CR6261 hIgG1
FI6 hIgG1
HA4 hIgG1
55 kDa
25 kDa
C
5J8 mIgG1
5J8 mIgG2a
F045-092 mIgG1
F045-092 mIgG2a
CR6261 mIgG1
CR6261 mIgG2a
FI6 mIgG1
FI6 mIgG2a
HA4 mIgG1
HA4 mIgG2a
55 kDa
25 kDa

Figure S4. SDS-PAGE of purified recombinant HA and VR analogous IgG variants. (A) Recombinant HA proteins from group 1 (A/New Caledonia/20/1999 (NC H1N1) and A/California/07/2009 (pH1N1)) and group 2 (A/Aichi/2/68 (H3N2A), A/Perth/16/2009 (H3N2P) and A/Netherlands/219/03 (H7N7). (B) F045-092, CR6261, FI6, and 5J8 human IgG1. (C) F045-092, CR6261, FI6, and 5J8 mouse IgG1 and mouse IgG2a.

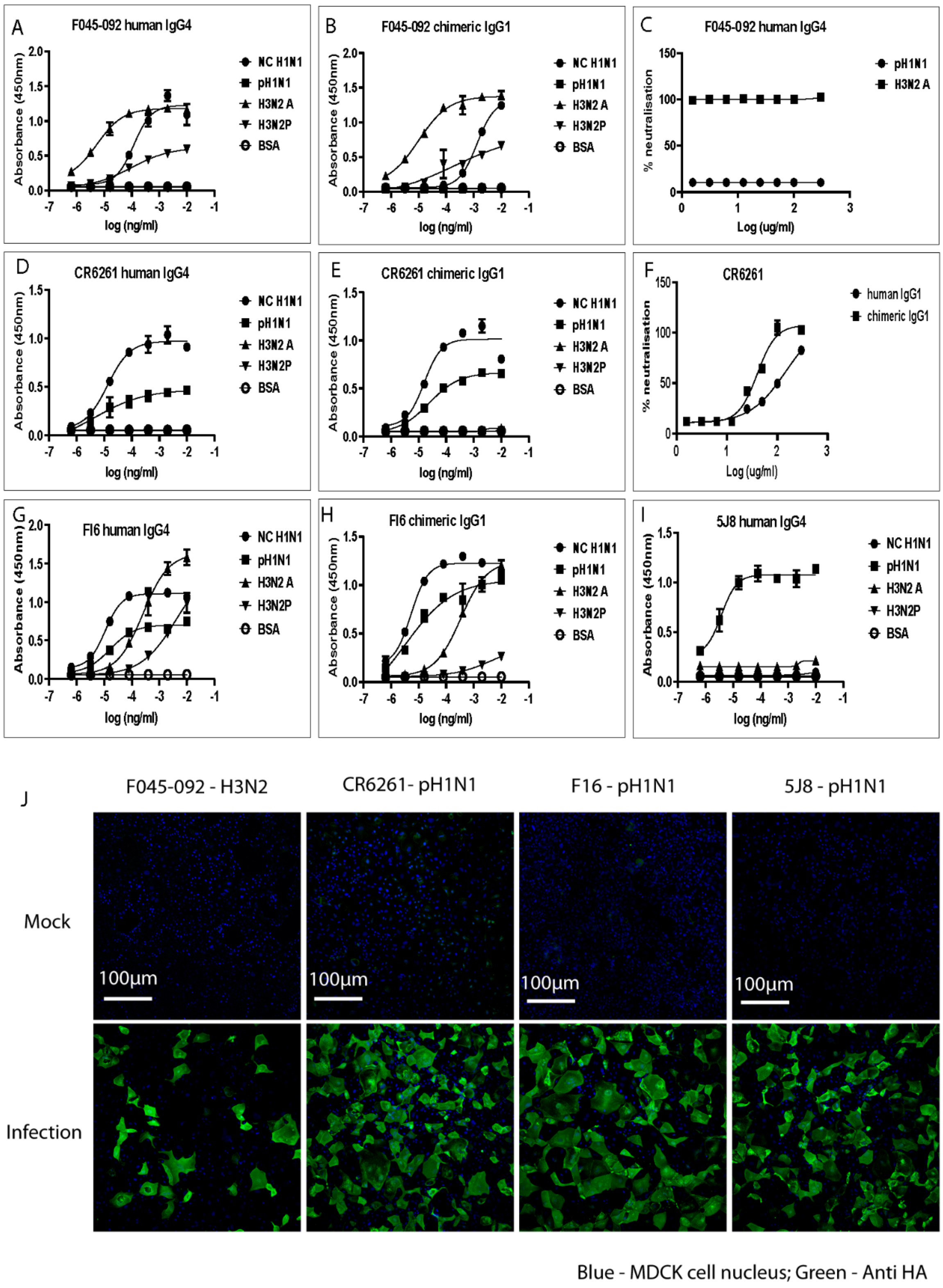
A
F045-092 human IgG4
B
F045-092 chimeric IgG1
C
F045-092 human IgG4
D
CR6261 human IgG4
E
CR6261 chimeric IgG1
F
CR6261
G
FI6 human IgG4
H
FI6 chimeric IgG1
I
5J8 human IgG4
Absorbance (450nm)
log (ng/ml)
% neutralisation
Log (ug/ml)
NC H1N1
pH1N1
H3N2 A
H3N2P
BSA
human IgG1
chimeric IgG1
J
F045-092 - H3N2
CR6261- pH1N1
F16 - pH1N1
5J8 - pH1N1
Mock
Infection
100µm
Blue - MDCK cell nucleus; Green - Anti HA

Figure S5. Biochemical characteristics of VR analogous IgG variants. (A) Binding profiles of F045-092 human IgG4 by ELISA. (B) Binding profiles of F045-092 human IgG1 with a mouse IgG1 hinge by ELISA. (C) Neutralization profiles of F045-092 human IgG4. (D) Binding profiles of CR6261 human IgG4 by ELISA. (E) Binding profiles of CR6261 human IgG1 with a mouse IgG1 hinge by ELISA. (F) Neutralization profiles for CR6261 human IgG1 and CR6261 human IgG1 with a mouse IgG1 hinge. (G) Binding profiles of FI6 human IgG4 by ELISA. (H) Binding profiles of FI6 human IgG1 with a mouse IgG1 hinge by ELISA. (I) Binding profiles of 5J8 human IgG4 by ELISA. (J) Binding profiles of F045-092, CR6261, FI6, and 5J8 human IgG1 by IFA using pH1N1 and H3N2A influenza viruses.
